# Bayesian modelling demonstrates clinically relevant heterogeneity in Tau PET patterns in Alzheimer’s disease

**DOI:** 10.1007/s00259-026-07868-5

**Published:** 2026-03-31

**Authors:** Ye Xia, Keith Johnson, Georges El Fakhri, Nicolas J. Guehl, Elsmarieke van de Giessen, Emma C. Coomans, Yolande A.L. Pijnenburg, Rik Ossenkoppele, Colin Groot

**Affiliations:** 1 Department of Radiology, Massachusetts General Hospital, Harvard Medical School, Boston, MA USA; 2 Department of Neurology, Alzheimer Center Amsterdam, Amsterdam UMC, Amsterdam Neuroscience, Amsterdam, Netherlands; 3 Amsterdam Neuroscience, Neurodegeneration, Amsterdam, the Netherlands; 4 Department of Radiology and Biomedical Imaging, Yale School of Medicine, New Haven, CT USA; 5 Department of Radiology and Nuclear Medicine, Amsterdam UMC, Amsterdam Neuroscience, Amsterdam, Netherlands; 6 Department of Biomedical Informatics and Data Science, Yale School of Medicine, New Haven, CT USA

**Keywords:** Alzheimer’s disease, Heterogeneity, Tau, PET, Latent Dirichlet Allocation

## Abstract

**Background:**

Traditionally, subgroups have been used to explore the effects of tau heterogeneity on cognition. However, categorization into rigid, exclusive subtypes, each with their own tau pattern, may overlook the fact that individual tau patterns are complex, and most individuals express features of multiple patterns. Mapping tau patterns in all their complexity has important clinical implications, as it may enable more accurate prognostication and support the development of personalized therapeutic strategies tailored to an individual’s unique tau profile.

**Methods:**

We applied a data-driven Bayesian model using Latent Dirichlet Allocation (LDA) to identify four covarying tau PET patterns in amyloid-positive individuals with symptomatic Alzheimer’s disease (AD) from the Amsterdam Dementia Cohort (ADC, *N* = 93, mean age = 65.3). The four latent tau spatial patterns identified via LDA were designated as follows: Limbic (Factor 1), characterized by predominant involvement of the limbic regions; Left TPC (Factor 2), centered on the left temporo-parietal cortex (TPC); Posterior (Factor 3), reflecting a posterior neocortical distribution; and MTL-sparing (Factor 4), showing relative sparing of limbic regions with diffuse neocortical tau deposition. Associations between individual loadings on each of these factors and cognitive domain scores were assessed using linear regression analyses. We then applied the ADC-derived model to an independent validation sample from the Alzheimer’s Disease Neuroimaging Initiative (ADNI, *N* = 162, mean age = 72.7) to extract individual factor loadings. Associations between factor loadings and contemporaneous cognitive performance were again tested using linear regression, and linear mixed-effects models were used to additionally explore associations with cognitive decline. All analyses were adjusted for age, sex, education, and clinical diagnosis.

**Results:**

In both the discovery and validation cohort, we identified distinct associations between tau factor loadings and cognitive performance. Higher loading on Factor 1: limbic tau was generally associated with relatively better baseline cognition and slower cognitive decline. Factor 2: left TPC tau was linked to worse baseline scores and faster decline in memory, MMSE and language scores but only in the ADNI cohort. Factor 3: posterior tau was associated with worse baseline MMSE in the ADC cohort and to worse visuospatial performance both cross-sectionally and longitudinally in the ADNI cohort. Factor 4: MTL-sparing tau was related to better longitudinal memory in the ADNI cohort.

**Conclusion:**

This data-driven approach identified overlapping tau patterns that relate to distinct cognitive domains. The findings highlight the value of continuous factor modeling in understanding heterogeneity in Alzheimer’s disease. Such a refinement of quantifying tau heterogeneity may improve patient stratification, refine prognostic accuracy, and ultimately guide the development of individualized treatment strategies.

**Supplementary Information:**

The online version contains supplementary material available at 10.1007/s00259-026-07868-5.

## Introduction

Alzheimer’s disease (AD) is a progressive neurodegenerative disorder characterized by the accumulation of extracellular amyloid-β (Aβ) plaques and intracellular neurofibrillary tangles composed of hyperphosphorylated tau. While Aβ deposition marks the onset of pathological changes, tau burden and regional distribution are more closely associated with clinical symptoms and cognitive decline [1, 2, 3]. Since 2013, tau pathology can be captured in vivo using tau-PET imaging [4]. Early tau-PET studies have corroborated post-mortem findings, confirming both the strong association between tau burden and cognition, and the domain-specific effects of regional tau deposition [4, 5, 6]. For example, tau accumulation in medial temporal regions is associated with memory impairment [7, 8], while tau in posterior cortices has been linked to visuospatial dysfunction [9] and left temporoparietal tau to language deficits [10]. These associations are not confined to atypical AD variants but are also observed across the broader AD spectrum [11]. To capture heterogeneity in tau distribution, recent studies have applied subtyping models that cluster individuals based on spatial tau-PET patterns [12, 13, 14]. A limitation of these studies is that they modelled heterogeneity as discrete, assigning individuals to mutually exclusive groups. This approach may overlook the continuous and overlapping nature of tau deposition across individuals [15].

An alternative approach to harnessing tau-PET data to examine neurobiological heterogeneity in AD is offered by a Bayesian modeling framework, Latent Dirichlet Allocation (LDA), which provides a numerical expression of an individual’s entire tau pattern. LDA might therefore be more suitable to represent the variable nature of tau distribution. Specifically, LDA can be applied to tau-PET by treating each individual’s tau-PET scan as a mixture of multiple underlying (i.e. latent) tau factors, allowing for spatially unconstrained, voxel-wise decomposition of tau patterns. While LDA has been previously applied to structural MRI data to identify latent atrophy patterns in AD and posterior cortical atrophy [16, 17], in this study, we apply LDA to identify latent tau patterns and explore how these patterns relate to domain-specific cognitive decline in AD. By allowing individuals to express multiple tau patterns without rigid subtyping, LDA offers a more nuanced understanding of tau heterogeneity and could thereby contribute to a more individualized approach to individualized prognoses. In clinical practice, this may allow clinicians to better anticipate which cognitive domains are most vulnerable in a given patient, supporting more precise prognostication and potentially informing personalized intervention strategies.

## Methods

### Participants

We included amyloid-positive individuals with symptomatic (i.e. mild cognitive impairment [MCI] or dementia) AD from two independent cohorts to identify (i.e. discovery cohort) and clinically validate (i.e. validation cohort) the LDA-derived tau PET patterns. To maximize the identification of relevant tau topographies, we selected the Amsterdam Dementia Cohort (ADC) as the discovery dataset. The ADC, established in 2000, is derived from the tertiary referral memory clinic of Alzheimer Center Amsterdam (Amsterdam University Medical Centers) [18]. The sample is characterized by a high degree of heterogeneity, partly resulting from a relatively young mean age of onset and a relatively high prevalence of atypical clinical presentations compared to other research cohorts. This diversity is critical for training the model to recognize all tau topographies that might present in AD. Subsequently, the ADNI cohort served as an external validation dataset. By projecting the factors learned from the heterogeneous ADC sample onto the ADNI data, we aimed to test the model’s generalizability and specificity in a more “typical” (late-onset) sample.

We selected 93 participants from the ADC who had undergone [^18^F]flortaucipir PET imaging with considerable variations in tau-PET patterns. Participants met the following inclusion criteria: (1) a clinical diagnosis of MCI or AD-type dementia based on established consensus criteria [19, 20], (2) confirmed amyloid positivity based on CSF assessment (cut-off value: <813 pg/mL; Innotest [21] or < 1,092 pg/mL; Elecsys [22]), and (3) evidence of sufficient cortical tau signal in a Hammers-atlas derived global composite region covering brain regions associated with Braak I–VI (standardized uptake value ratio [SUVR] ≥ 1.1; derived from the mean + 1.65 SDs in amyloid-negative controls [see below]) [23], ensuring adequate tau-PET signal for LDA modeling. This resulted in a sample of 95 participants out of the 182 available at time of selection. We also included 35 cognitively unimpaired, amyloid-negative controls from the ADC (age: 64.9[7.6], sex 18(51%) female, MMSE: 29.0[1.4]) [24], this group served as the reference to standardize participants’ tau-PET signal (see “Bayesian model” section below). In ADC, there were 2 individuals included that adhered to criteria for the behavioral variant of AD, 3 with the logopenic variant primary progressive aphasia, and 6 with posterior cortical atrophy.

For the validation cohort, 162 participants of the available 826 participants at time of selection were included from the Alzheimer’s Disease Neuroimaging Initiative (ADNI) database (https://adni.loni.usc.edu/) based on a clinical diagnosis of MCI or dementia, amyloid-positivity (amyloid-PET; Centiloid > 20 [25]) and the same SUVR > 1.1 threshold as for the ADC cohort but in a global cortical region based on the Desikan Killiany encompassing the same regions as the Hammers-derived Braak I–VI region in the ADC cohort. Since LDA works in a voxel-wise manner, slight differences in global tau masks used to select individuals with sufficient tau has minimal impact on results. From ADNI, we further selected a group of 193 amyloid-negative cognitively unimpaired individuals (age = 68.5[6.2], sex, 102 [53%] female, MMSE = 29.1[1.1]) who served as the reference to standardize participants’ tau-PET signal (see “Bayesian model” section below). ADNI was selected as the validation cohort to clinically validate the LDA-derived tau factors due to the availability of extensive neuropsychological follow-up in this dataset, allowing us to test the predictive effects of regional tau on longitudinal domain-specific cognitive decline.

To ensure a robust biological definition of Alzheimer’s disease, all included participants in both cohorts were required to be amyloid-positive. The method of ascertainment was selected to maximize data availability and consistency within each cohort. In the Discovery Cohort (ADC), amyloid positivity was determined using CSF biomarkers, as these were available for all participants at diagnosis (amyloid-PET was restricted to a small subset). In contrast, for the Validation Cohort (ADNI), amyloid positivity was defined solely using amyloid-PET, which was available for the majority of participants.

### Standard protocol approvals, registrations, and patient consents

Written informed consent was obtained from all participants from the ADC at Amsterdam UMC, study procedures were in accordance with the Declaration of Helsinki and approved by the institutional review board of the Amsterdam UMC (REC 2017.315). Data used in the preparation of this article were obtained from the Alzheimer’s Disease Neuroimaging Initiative (ADNI) database (adni.loni.usc.edu) and informed consent was obtained from all participants or their authorized representatives at each site at the time of enrollment, in accordance with the Declaration of Helsinki. The ADNI study was approved by the institutional review boards of all participating institutions.

### Cognitive measurement

In ADC, a comprehensive neuropsychological test battery was categorized into 5 cognitive domains (memory, attention, language, executive functioning, and visuospatial ability). Memory was assessed with the Dutch version of the Rey Auditory Verbal Learning Test (RAVLT), the immediate and delayed recall conditions, as well as part A (two trials) of the Visual Association Test (VAT). Language was measured with the naming condition of the VAT and the animal fluency test (60-second). We assessed executive functioning with the letter fluency test, Stroop card 3, and digit span backward. Visuospatial ability was assessed with the following tests from the Visual Object and Space Perception battery (VOSP): fragmented letters, dot counting, and number location. Scores that were based on the time taken to complete the test (i.e., Stroop and TMT) were inverted so that lower scores indicated worse performance. All raw test scores were then converted into *z* scores based on the mean and SD from independent normative data from 440 cognitively unimpaired (CU) amyloid-β-negative participants from the ADC (mean age 59.9 years [*SD =* 6.05], 40% female)^1^. The *z*-scores of tests within each domain were averaged to generate the composite scores. These composite scores were only calculated when 2 or more tests were available with each domain (*N* = 89 for Memory, *N* = 89 for Executive Functioning, *N* = 85 for Language and *N* = 80 for Visuospatial Functioning).

Cognitive performance in the ADNI cohort was assessed using standardized and validated composite scores developed within ADNI to quantify functioning across four cognitive domains: memory (ADNI-MEM [26]), executive function (ADNI-EF) [27], visuospatial abilities (ADNI-VS) [28], and language (ADNI-LAN [28]). Baseline data was available for all ADNI participants across all domains except Executive Functioning (*N* = 161). Of the 162 ADNI participants, 125 had longitudinal follow-up available (mean follow-up time = 2.4[1.3] years).

The Mini-Mental State Examination (MMSE) was used to assess global cognitive impairment in both cohorts.

### Image acquisition and processing

In the ADC discovery cohort, [^18^F]flortaucipir PET scans were acquired on an Ingenuity time of flight PET/CT scanner (Philips Medical Systems, Best, The Netherlands), as previously described [29, 30]. Briefly, a low-dose CT scan was performed prior to acquisition for attenuation correction. PET data were acquired 80–100 min post injection of [^18^F]flortaucipir (target dose: 185 MBq). PET images were acquired in 3D mode and reconstructed with the RAMLA algorithm (128 × 128 × 90 matrix, 2 × 2 × 2 mm³ voxels). Standard corrections were applied, including attenuation, scatter, randoms, decay, and dead-time correction. High-resolution T1-weighted MR images acquired a maximum of one year from the tau-PET scan were co-registered to PET using VINCI software. ROIs were delineated on MRI using the Hammers atlas [31, 32] and projected onto the co-registered PET images using PVElab, whereupon voxel-wise SUVR images using the whole cerebellar gray matter as the reference region were computed using an in-house pipeline [33]. Subsequently, the MRI images were normalized to MNI standard space using Statistical Parametric Mapping (SPM). The resulting normalization parameters were then applied to the co-registered tau-PET images to bring them into the same MNI standard space [34].

For the ADNI cohort, [^18^F]flortaucipir (target dose: 370 MBq) PET scans were acquired 80–100 min post-injection and processed following the standardized tau PET protocols established by the ADNI PET core (https://adni.loni.usc.edu/methods/pet-analysis/ ), ensuring methodological consistency with prior large-scale tau PET studies using the ADNI dataset [35, 36]. Core steps included motion correction, co-registration to MRI, spatial normalization to MNI template space, and SUVR computation using cerebellar gray matter as the reference region (https://adni.loni.usc.edu/methods/pet-analysis/ ).

For both the discovery and validation cohort, tau-PET SUVR images were converted into W-score maps (i.e., control-normalized and covariate-adjusted *z* scores) by performing voxel-wise standardization to each cohort’s control group, regressing out the effects of age, sex, and global tau-PET SUVR. The latter covariate was added to ensure that covariance in tau-PET patterns detected by the LDA model represented differences in spatial patterns of tau, rather than differences in disease stage. This voxel-wise normalization step ensures that the input data for the LDA decomposition represented tau burden relative to a healthy control population. The resulting W-maps are voxel-wise covariate-adjusted tau-PET SUVR images. In accordance with methodological procedures for the application of LDA to structural MRI [37], voxels not likely to contribute to biological disease variation were excluded, and so tau-PET images were deprecated at SUVR = 1.

### Bayesian model

We applied a Bayesian modeling framework called Latent Dirichlet Allocation (LDA) to identify latent tau patterns that co-vary across individuals [16]. Originally developed to extract topics in textual corpora, LDA was previously adapted for neuroimaging to detect covarying patterns of gray matter voxel intensities on structural MRI [16]. By applying LDA to tau-PET, this approach allows for a data-driven, voxel-wise decomposition of tau patterns. In the model, each tau PET scan is treated as a collection of voxels that can be assigned to multiple latent tau factors(K), which are spatially unconstrained and might overlap. Instead of categorizing individuals into rigid subtypes, LDA allows each participant to express each tau factor to a certain extent, and the combination of these expressions (together called factor compositions) represent the entire tau pattern of that individual.

Specifically, a variational Bayesian inference approach, a variational expectation–maximization (VEM) algorithm estimates a probability for each given voxel to belong to a latent factor (Pr[voxel|factor]). Then, for each individual and for each factor, the probability that a factor contributes to an individual’s tau PET scan (Pr[factor|scan]) is estimated. We used symmetric Dirichlet priors on participant-level factor compositions and factor-level voxel distributions, with fixed hyperparameters following the standard LDA implementation described by Zhang et al. (2016) [2]. The latent tau factors (Pr[voxel|factor]) are represented as voxel-wise probabilistic maps that show the anatomical location of the tau factors, while Pr[factor|scan] is a quantitative measure of the relative contribution of each factor to an individual’s tau burden. For example, in a four-factor model (K = 4), a subject might exhibit e.g., 10% factor 1, 20% factor 2, 50% factor 3, and 20% factor 4, with all factor expressions summing to 100%. This composition indicates the relative proportion of different tau patterns to an individual’s total tau-PET signal rather than the magnitude of tau burden, which means that the factor compositions are reflective of tau heterogeneity rather than stage. The number of factors that LDA finds the solution for is supervised, and to determine the optimal number of factors, we ran all factor-solutions from K = 2 to K = 6. The optimal number of latent factors was then determined using a likelihood-based elbow criterion, defined as the smallest K after which relative improvements in model fit fell below a predefined tolerance and decreased monotonically, indicating diminishing returns. Each model was run using 8 random initializations and correlations between the initialization with the best model fit and the others were assessed.

We selected the LDA framework specifically for its advantages over traditional spatial clustering approaches in two key aspects: The first one being brain region agnosticism. Unlike traditional approaches that impose rigid a priori anatomical boundaries, LDA is a voxel-wise approach. It does not rely on pre-defined regions of interest, allowing the model to detect naturally occurring tau covariance networks that may cross or occupy only parts of standard anatomical boundaries. The second advantage is probabilistic (soft) membership, both of individuals to factors and of voxels to factors. Crucially, unlike ‘hard’ clustering methods where an individuals or voxel is assigned to a single subgroup or cluster (i.e., binary membership), LDA allows for probabilistic (soft), mixed membership. This means that an individual can express multiple factors with varying probabilities (e.g., a voxel in the precuneus might load onto a ‘Posterior’ factor and onto a ‘Diffuse’ factor), and a single voxel can thereby contribute to multiple latent factors simultaneously. This property is particularly biologically plausible for neurodegenerative disease, where individuals generally show pathology across multiple networks and brain areas, and brain regions are often hubs involved in multiple overlapping pathological networks.

### Statistical analysis

Statistical analyses to assess the power of the LDA-derived factor loadings to predict baseline and longitudinal cognition were conducted using R (version 4.4.1). We performed linear regression (package: lm) analyses to assess associations between tau factors and baseline cognition. We ran linear mixed effects analyses (package: lme4) to assess associations between tau factors and longitudinal cognition (ADNI validation cohort only). All models were adjusted for age, sex, education and clinical disease stage (MCI/dementia). We modeled each factor individually to assess its relationship with cognitive performance and a negative association between a tau factor (X) and a cognitive domain (Y) implies that individuals with higher tau burden in regions associated with factor X relative to the other factors have worse performance in domain Y. Conversely, a positive association between factor X and domain Y indicates that a pattern that is characterized by high tau in regions associated with factor X (rather than the other factors) results in better measures on domain Y. Sensitivity analyses exploring associations between age, sex, APOEε4 and factor loadings are performed using general linear models.

Significance threshold for all analyses was set as α = 0.05, without correction for multiple comparisons.

### Data availability

The full code and documentation for the Bayesian modeling approach used here is publicly available at https://github.com/ThomasYeoLab/CBIG/tree/master/stable_projects/disorder_subtypes/Zhang2016_ADFactors. Anonymized data used in the present study may be available upon request to the corresponding author.

## Results

Demographic and clinical characteristics of the ADC discovery and ADNI validation cohorts are displayed in Table 1. Compared to the ADNI cohort, participants in the ADC cohort were significantly younger (65.3 ± 7.6 vs. 72.3 ± 7.2 years; *p* < 0.001), had fewer years of education (12.3 ± 3.0 vs. 15.7 ± 2.4 years; *p* < 0.001), and showed higher whole-brain tau-PET SUVR (1.5 ± 0.4 vs. 1.4 ± 0.3; *p* = 0.009). No significant differences were observed between cohorts for sex distribution (*p* = 0.364) or APOEε4 carrier status (*p* = 0.162).

**Table 1:** Demographic characteristics and whole-brain Tau-PET SUVR in the discovery (ADC) and validation (ADNI) cohorts

	ADC	ADNI	*p*
*N*	93	162	
Age (years)	65.26 ± 7.58	72.65 ± 7.22	0.000
Female N (%)	43 (46.2%)	85 (52.5%)	0.364
Education (years)	12.26 ± 2.99	15.68 ± 2.42	0.000
APOEε4 Carrier N (%)	65 (73.0%)	104 (64.2%)	0.162
Whole-brain Tau-PET SUVR	1.50 ± 0.36	1.38 ± 0.30	0.009

*ADNI* Alzheimer’s Disease Neuroimaging Initiative, *APOEε4* Apolipoprotein E4, *SUVR* Standardized Uptake Value Ratio. Age is presented as mean ± standard deviation (SD). Sex is reported as the number and percentage of female participants. APOE4 carrier status was defined as having at least one ε4 allele (i.e., genotypes including one or two ε4 alleles). Education was recorded as years of formal education. Whole-brain tau-PET SUVR reflects the mean standardized uptake value ratio across the cortex. P-values represent comparisons between ADC and ADNI cohorts, calculated using independent t-tests for continuous variables and Fisher’s exact tests for categorical variables

### Factor compositions in the discovery and validation cohorts

**Fig. 1:**
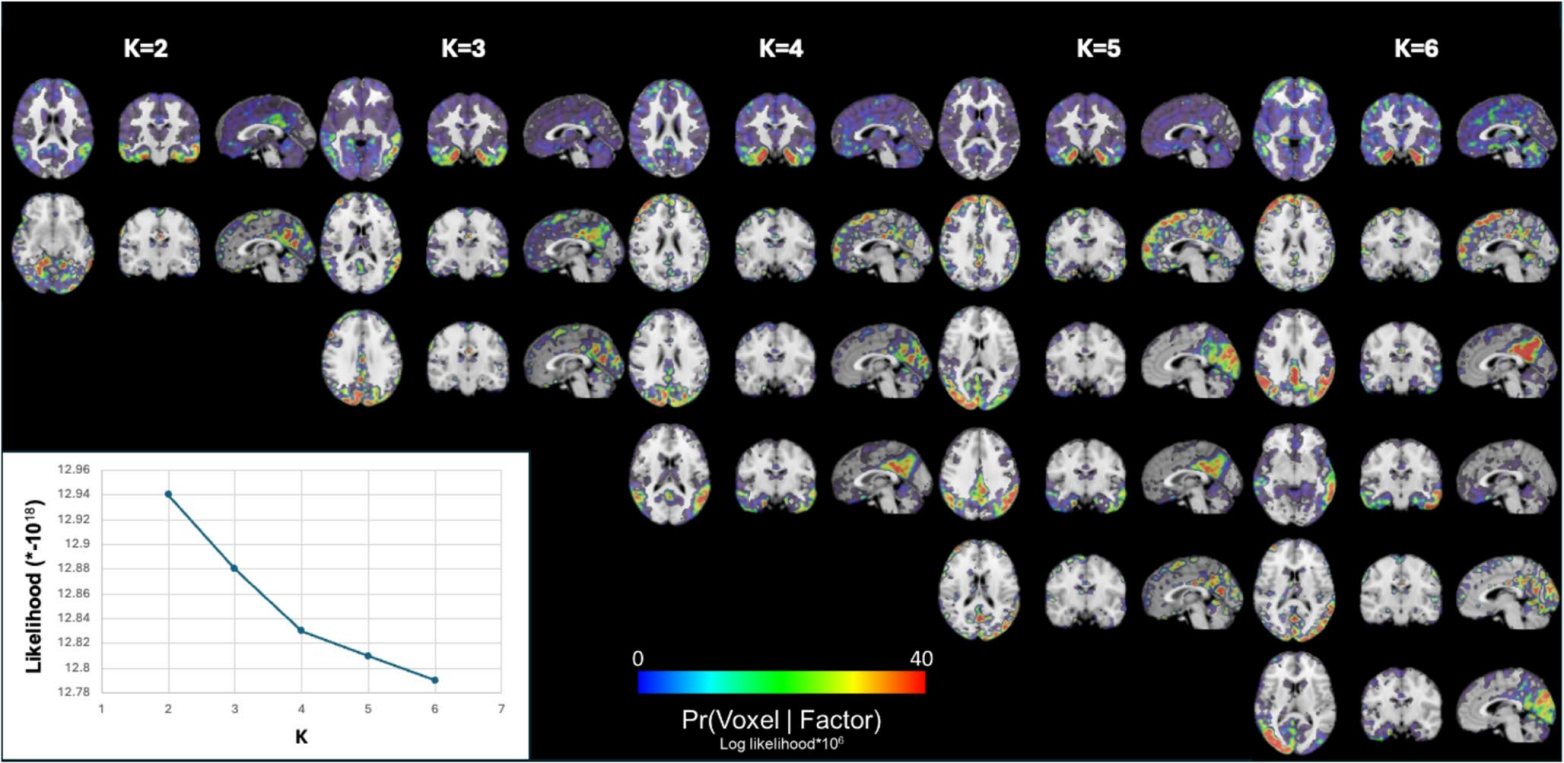
Latent spatial factors derived from decompositions with K = 2 through K = 6 components. Each column corresponds to a specific value of the supervised number of factors in the LDA analyses (K), increasing from left to right. Spatial patterns of the factors are shown across different anatomical orientations: transverse plane, coronal plane, and sagittal plane. Colored overlays represent the probabilities of each voxel belonging to a factor (Pr[voxel|factor]), mapped onto a standard MNI template. Images are displayed in radiological convention. The visualization displays the stability and progressive fractionation of tau topography as the number of components increases. The bottom-left panel shows the overall model log-likelihood for each value of K (K = 2 to K = 6), showing model fit across decompositions and highlighting a visual “elbow” in model fit increase with model complexity at K = 4. A data-driven justification for our choice of selection K = 4 as the optimal model is outlined in Supplemental Table 1

**Fig. 2:**
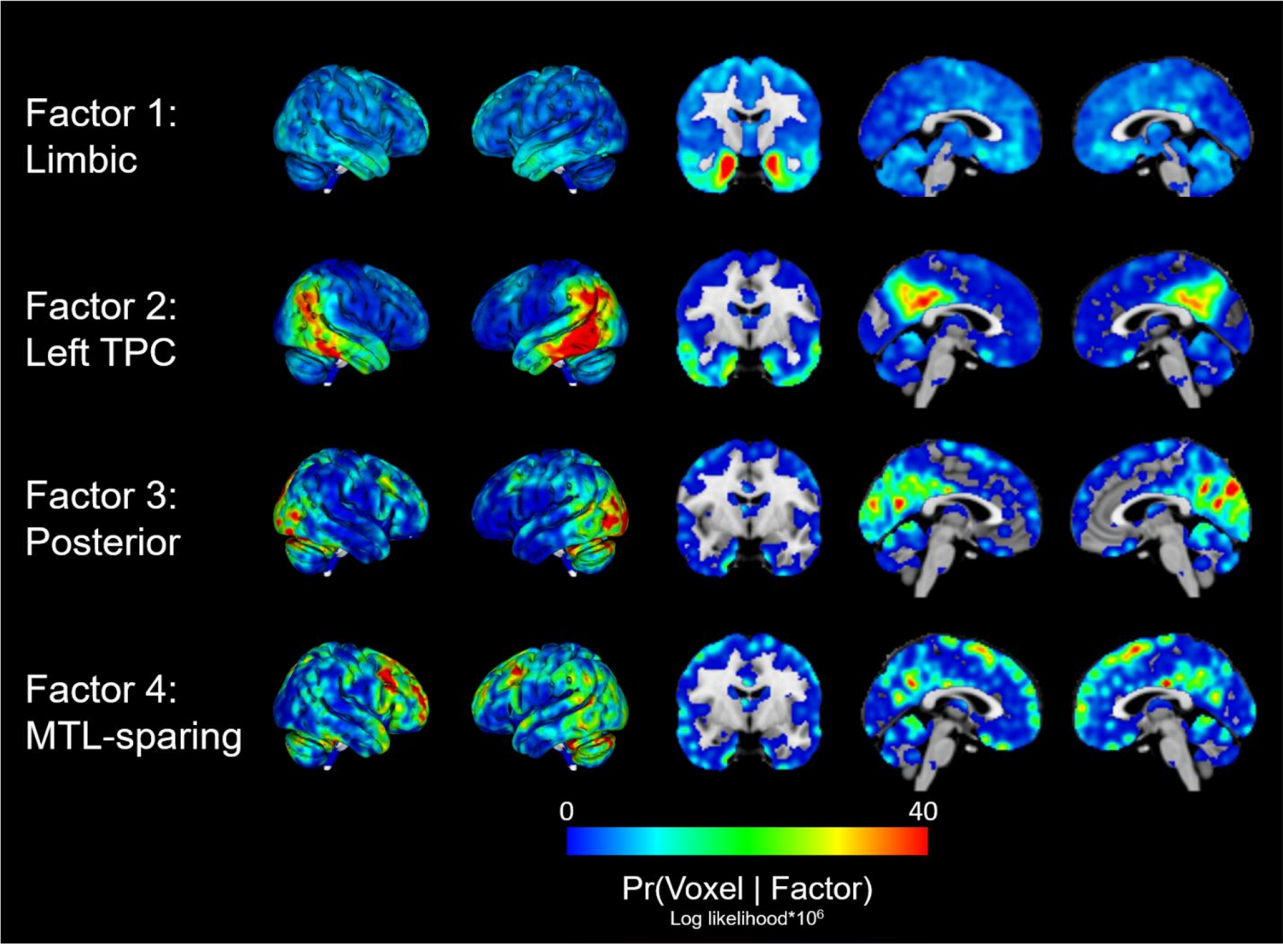
Voxel-wise probability maps across latent tau factors for the four-factor solution. The colours indicate the probability of each voxel to belong to each of the factors (Pr[voxel|factor). Factors were named in accordance with the anatomical regions with the highest probability of belonging to each factor. TPC-temporoparietal cortex, MTL-medial temporal lobe

In the ADC discovery cohort, we applied factor decomposition models across a range of factor numbers (K = 2 to K = 6; Fig. 1). As the supervised number of factors increased from K = 2 to K = 6, the resulting spatial patterns exhibited a clear hierarchical structure. Factors identified at lower K were preserved or subdivided at higher K values, indicating stable and nested decomposition. Relative improvements in log-likelihood decreased steadily with increasing K, with gains falling below 0.25% beyond K = 4. This pattern indicates a saturation of model fit and supports selection of a four-factor solution (Fig. 1; Supplemental Table 1). The 8 random initialization of the four-factor solution showed similar model fits and the correlations between the initializations was high (*r* > 0.9; Supplemental Figs. 1 and 2). The four-factor solution yielded four distinct but partly overlapping tau patterns, which were named according to the anatomical location of voxels most likely to belong to each factor: Limbic (encompassing medial temporal lobe and other limbic structures; Factor 1), Left TPC (stretching across the temporo-parietal cortex [TPC] and left-lateralized; Factor 2), Posterior (encompassing occipital and posterior parietal regions; Factor 3), and MTL-sparing (widespread tau deposition across multiple cortical regions; Factor 4; Fig. 2).

**Fig. 3:**
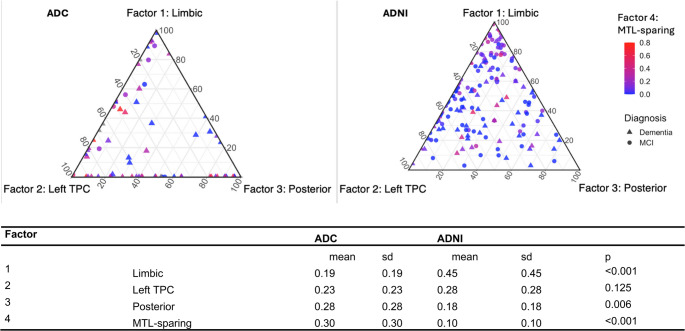
Factor compositions.The ternary plots illustrate how individual tau factor compositions are distributed across three spatial patterns: limbic, left temporo-parietal cortex (TPC), and posterior. The fourth factor, representing medial temporal lobe (MTL)-sparing, is shown as a color gradient from blue (low) to red (high). Each point represents one participant. Triangles indicate dementia. Circles indicate mild cognitive impairment (MCI)

Figure 3 illustrates the distribution of factor loadings in the ADC discovery cohort (left) and ADNI validation (right) cohort, showing the inter-individual variability in tau deposition patterns. Each individual’s tau burden is decomposed into four factors representing the individual’s spatial components. In the ADC cohort, participants exhibited factor compositions that clustered toward the edges of the ternary plots, indicating distinct individual tau-patterns that were associated with one factor (Fig. 3). For ADNI, the factor compositions clustered toward the middle of the plot, with a greater number of cases occupying regions of the triangle corresponding to mixed or balanced factor composition. Descriptive statistics (mean and standard deviation) for each of the four tau factors: Limbic (Factor 1), Left TPC (Factor 2), Posterior (Factor 3), and MTL-sparing (Factor 4) are provided in Fig. 3 for both the ADC and ADNI cohorts. Posterior and MTL-sparing factor loadings were higher in ADC compared to ADNI (both *p* < 0.01) and limbic factor loading was higher in ADNI (*p* < 0.001; Fig. 3).

### Associations between factor loading and cognition

#### Discovery

In the ADC cohort, higher Limbic (Factor 1) loading was associated with better executive functioning (β[SE] = 0.31[0.13], *p* = 0.02), language (β[SE] = 0.27[0.13], *p* = 0.04), and MMSE scores (β[SE] = 0.23[0.12], *p* = 0.05). Higher Left TPC (Factor 2) loading was associated with better baseline visuospatial functioning (β[SE] = 0.25[0.10], *p* = 0.02). Higher Posterior (Factor 3) loading was associated with worse MMSE scores (β[SE]=–0.27[0.11], *p* = 0.01).

#### Validation

Limbic (Factor 1) factor loading was positively associated with all cognitive domains at baseline, memory (β[SE] = 0.26[0.05], *p* < 0.001), executive functioning (β[SE] = 0.34[0.06], *p* < 0.001), language (β[SE] = 0.21[0.06], *p* < 0.001), visuospatial ability (β[SE] = 0.24[0.07], *p* < 0.001), and MMSE (β[SE] = 0.19[0.07], *p* = 0.01). Similarly, Limbic factor loading was positively associated with longitudinal trajectories in memory (β[SE] = 0.11[0.02], *p* < 0.001), executive functioning (β[SE] = 0.11[0.02], *p* < 0.001), language (β[SE] = 0.15[0.03], *p* < 0.001), visuospatial ability (β[SE] = 0.09[0.04], *p* = 0.01), and MMSE (β[SE] = 0.24[0.03], *p* < 0.001). Indicating that a tau pattern that is limbic-predominant (rather than neocortical) relates to better clinical measures. The Left TPC (Factor 2) was negatively associated with baseline memory (β[SE]=-0.21[0.05], *p* < 0.001), language (β[SE]=-0.13[0.06], *p* = 0.03), and MMSE (β[SE]=-0.14[0.06], *p* = 0.03). Left TPC factor loading was also significantly negatively associated with longitudinal decline in memory (β[SE]=-0.12[0.02], *p* < 0.001), executive functioning (β[SE]=-0.09[0.02], *p* < 0.001), language (β[SE]=-0.13[0.03], *p* < 0.001), and MMSE (β[SE]=-0.20[0.03], *p* < 0.001). These findings suggest that a pattern with prominent tau deposition in the left TPC is associated with worse cognitive outcomes across various domains. Posterior (Factor 3) factor loading was negatively associated with baseline executive functioning (β[SE]=-0.24[0.06], *p* < 0.001) and visuospatial ability (β[SE]=-0.17[0.07], *p* = 0.01). Longitudinally, it was also negatively associated with visuospatial ability (β[SE]=-0.11[0.05], *p* = 0.02), indicating that greater posterior tau involvement predicts decline in visuospatial function. Finally, the MTL-sparing (Factor 4) was not significantly associated with baseline cognition but showed a positive longitudinal association with memory (β[SE] = 0.04[0.02], *p* = 0.01). Effect size estimates with their confidence intervals for all baseline and longitudinal effects are displayed in Supplementary Table 2.

### Sensitivity analyses

We also explored the associations between tau factor loadings and demographics (age, sex, APOEε4). In the discovery cohort (ADC), age showed a significant negative association with the Posterior factor (β = −0.014, *p* < 0.001), indicating lower factor loadings in older individuals, while the Limbic factor showed a positive association with age (β = 0.018, *p* < 0.001). A significant sex difference was observed for the Limbic factor (β = −0.122, *p* = 0.025), suggesting lower Limbic factor loadings in males. No significant associations were observed with APOEε4 carrier status and any factor loading (all *p* > 0.05). In the validation cohort (ADNI), no significant associations were observed between factor loadings and age, sex, or APOE ε4 status (all *p* > 0.05; Supplemental Table 3).

**Fig. 4:**
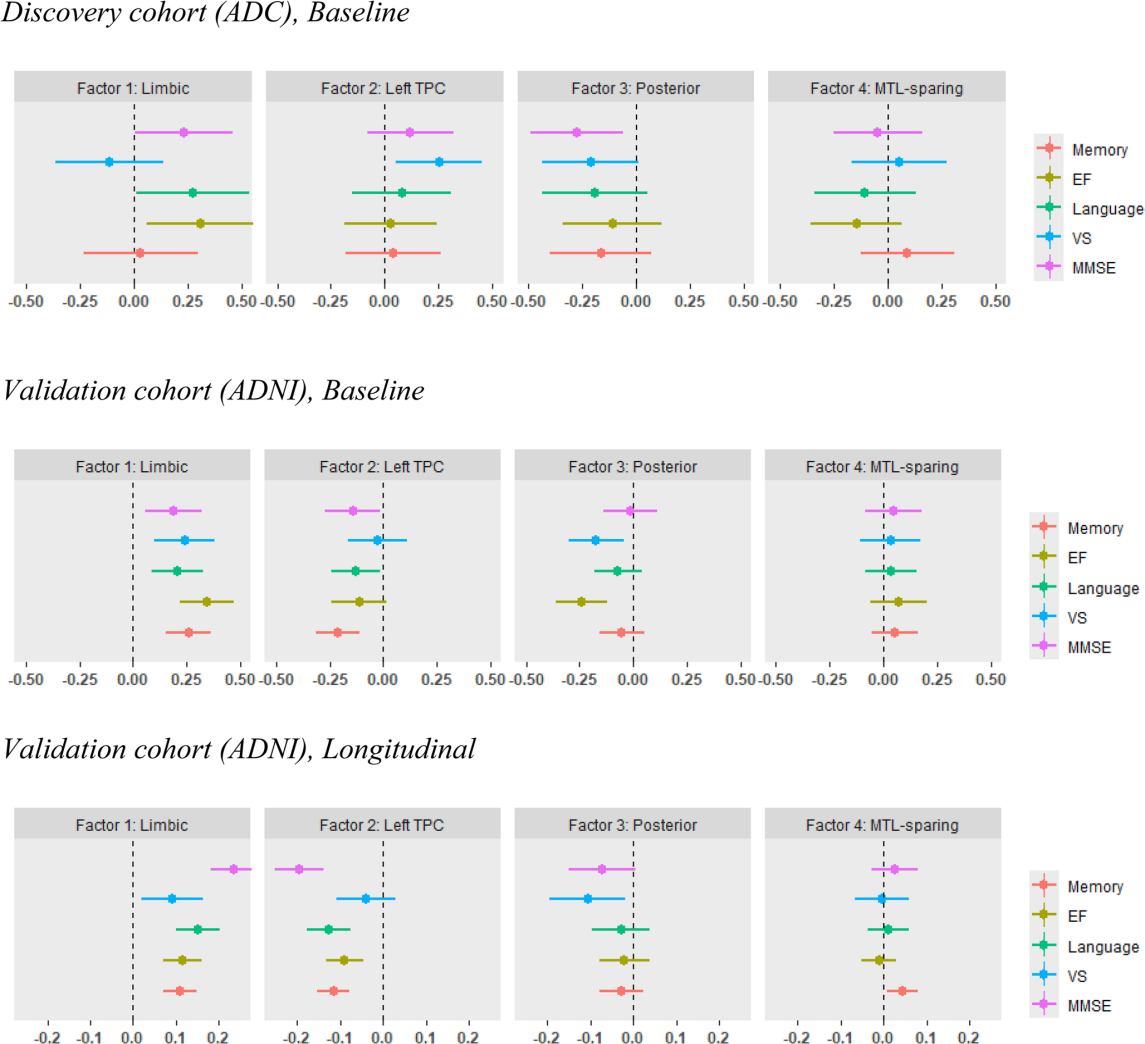
Associations between factor loadings and cognition. Associations between tau factor loadings and cognitive performance at baseline and longitudinally. Each plot presents standardized beta estimates and 95% confidence intervals from linear regression analyses (for baseline effects) or linear mixed-effects models (for longitudinal effects). Baseline effects were inferred from the fixed effect of factor on cognition, while longitudinal effects were inferred from factor*time=cognition interaction effects. Models were corrected for age, sex, education and syndromic diagnosis. *TPC *Temporoparietal cortex, *MTL *Medial temporal lobe

## Discussion

In this study, we identified four distinct yet partially overlapping tau factors among participants with symptomatic Alzheimer’s disease (AD) using a data-driven Bayesian modeling approach and applied these factors to examine relationships with cognition in two independent cohorts. We identified a Limbic (Factor 1), Left temporo-parietal cortex (TPC, Factor 2), Posterior (Factor 3), and MTL-sparing (Factor 4) factor, and each of the factors was associated with cognitive profiles and trajectories. The medial-temporal factor generally related to better performance and more gradual decline across all domainse. In the validation cohort the TPC factor was related to worse measures across a range of cognitive measures and the posterior factor showed an association with executive deficits and visuospatial ability. Notably, the MTL-sparing factor showed no associations with any cognitive domain at baseline, likely because it reflects a non-specific aspect tau burden rather than focal network-level pathology, and therefore does not appear to contribute to clinical or cognitive heterogeneity. ”Taken together, these findings highlight that clinically relevant tau-PET patterns can be characterized without rigid subtyping while allowing individuals to express multiple tau patterns to varying degrees. By refining how tau heterogeneity is mapped, this approach has the potential to improve patient stratification, enhance prognostic precision, and inform the development of personalized treatment strategies.

Until recently, the prevailing theory to describe tau pathological spread has been that of Braak and Braak [38], even though this model does not adequately capture the diversity of tau patterns observed between cases. The study of tau heterogeneity has been greatly facilitated by tau-PET, which allows the capture of spatial spreads of tau in-vivo [39]. In recent years, efforts have been underway to leverage tau-PET in order to capture and classify tau heterogeneity, mostly by using clustering approaches to produce subtypes, using tau-PET alone or in combination with other modalities [40, 41, 42]. These clustering-based studies, including work by Jeon [41] et al., have consistently identified tau deposition subtypes aligning with differences in cognitive presentation and atrophy distributions. Similarly, SuStaIn and event-based modeling frameworks have described subtype-specific temporal progression trajectories using amyloid, tau, or structural data [43, 44]. Other approaches, including graph-based [45] and hybrid supervised–unsupervised models [46], have uncovered biologically and behaviorally meaningful subgroups by combining multimodal features. The convergence of findings across modeling approaches highlights several consistent themes: AD pathology is multidimensional, its manifestation across individuals is variable, and this variability has meaningful consequences for the clinical manifestation of AD. Taken together, the differences in methods, whether discrete versus continuous, spatial versus temporal, or supervised versus unsupervised largely reflect different emphases rather than contradictions.

Across studies, the most robust findings regarding patterns of tau have been a posterior pattern, a (left) temporal pattern, a limbic pattern, and an MTL-sparing pattern. This was most notably shown in a study implementing SuStaIn to map heterogeneity in tau patterns [43]. Our study aligns with these prior works identifying co-varying tau topographies and these results are also largely in line with what has been found using similar approaches to capture atrophy subtypes on structural MRI [47, 11, 48]. These varying subtypes also correspond to areas that are predominantly affected in the most common atypical variants of AD; a posterior tau pattern in posterior cortical atrophy, and a (left) temporal pattern in the logopenic variant of primary progressive aphasia [49], which could be regarded as the extremes of the tau phenotypic variability spectrum. Furthermore, the MTL-sparing and limbic-predominant patterns are reminiscent of reported tau patterns in early and late-onset AD, respectively [50, 51]. Complementing discrete clustering or staging frameworks, our dimensional approach models the expression of tau patterns as overlapping and continuous, offering a biologically plausible view of tau heterogeneity. Our method differs in another key way from subtyping approaches: it allows individuals to express multiple tau patterns to varying degrees, rather than assigning them to a single subtype based on a dominant pattern. Indeed, when examining individual expressions of these factors, we found that most participants exhibited contributions from multiple factors, rather than predominantly expressing a single factor. This suggests that most individuals exhibit tau deposition across multiple brain regions, rather than focal involvement restricted to a single area, showing the potential added value of dimensional approaches in capturing tau heterogeneity. By estimating continuous factor loadings, LDA also allows for a more granular decomposition, enabling the mapping of the full spectrum of tau heterogeneity.

It has been well-established that regional spread of tau influences clinical manifestations of AD. This is exemplified by the tau patterns observed in atypical variants of AD, each with their extreme clinical phenotype [47]. But this clinicopathological correlation is also observed among subgroups of individuals within the “typical AD” spectrum [52]. We show that continuous tau loadings are differentially associated with distinct cognitive domains; posterior factor loading was associated with baseline executive and baseline and longitudinal visuospatial functioning, left TPC (Factor 2) factor loadings with baseline and longitudinal memory, language and global cognition (MMSE), and Limbic (Factor 4) factor loading was broadly associated with better cognitive outcomes, although the posterior and Left TPC results were largely based on results in the validation cohort alone. We can, however, distill two main trends from these results; (1) limbic-predominant tau is associated with better outcomes and neocortical tau with worse outcomes, and (2) among the neocortical tau patterns posterior tau is specifically related to visuospatial function while TPC tau is broadly associated with cognitive domains impaired in AD, except visuospatial functioning. This domain-specificity provides support for the utility of dimensional models in capturing tau-cognition relationships, which could aid diagnostic and prognostic procedures or provide handholds for investigations into regional susceptibility to tau.

The mechanisms underlying this regional susceptibility to AD pathology, and specific vulnerability to impairment in specific cognitive domains, remain largely elusive. Possible contributors could be developmental, structural, or genetic factors. For example, individuals with posterior cortical atrophy or logopenic variant primary progressive aphasia are more likely to report learning difficulties in childhood, possibly reflecting early neurodevelopmental anomalies that may predispose certain cortical networks to later tau accumulation and/or result in vulnerabilities to specific cognitive impairments [53]. From a genetic perspective, *APOE*ε4 has been associated with memory impairment and with increased medial temporal tau pathology [54]. However, direct assessments of *APOEε*4’s effects on tau-PET have revealed only modest and regionally diffuse differences, suggesting that *APOE* likely plays a secondary role in shaping tau topography [55, 56]. The effects of learning difficulties and genetics on regional variations in tau-PET are an active area of research and their effects on LDA-derived tau factors will need to be examined in future studies.

### Strengths and limitations

The study has several notable strengths, including the clinical validation of our LDA-factors in an independent sample with extensive follow-up available, which highlights the clinical utility and generalizability of the identified tau patterns. This study also has several limitations. Tau PET imaging has inherent constraints, particularly in detecting low tau burden. In those individuals with low tau burden, the model assigns factor loadings based on limited signal availability, which is partly related to noise, possibly leading to lower accuracy of mapping signal related to tau. This limitation represents a broader sensitivity issue in tau PET imaging. Another limitation is the cross-sectional nature of the tau PET data, restricting our ability to examine longitudinal stability of tau factor compositions. Longitudinal studies are required to determine whether individuals maintain stable tau factor compositions. Another potential limitation is the differences between the discovery and the validation samples, for example the differences in mean age. The selection of the ADC cohort for the discovery phase was, however, intentional. The ADC cohort, partly attributed to the lower average age, provides a broader representation of tau variability. This diversity was critical for the model to successfully characterize all tau topographies. Conversely, the ADNI cohort allowed us to validate the model’s generalizability in a more “typical” late-onset sample. The difference in cohorts also resulted in differences in factor compositions, with the model assigning higher limbic loadings in ADNI. We interpret this distribution as a confirmation of the model’s ability to detect tau topography as it presents itself, given that ADNI is largely comprised of typical late-onset amnestic phenotypes. Importantly, while less prevalent, individuals with high neocortical loadings were still detected within ADNI. This confirms that our approach using a heterogeneous discovery sample which yields a model capable of detecting atypical patterns, as well as correctly characterizing typical presentations, whenever they occur. While differences across cohorts remain, they are likely driven in part by variations in cognitive test batteries, language-related biases, and differing approaches to domain score construction (z-score–based composites in ADC versus latent factor–derived scores in ADNI). Lastly, although we aimed to adjust for differences in disease stage when examining tau heterogeneity, and found no clear tendency for MCI or dementia patients to load more heavily on a specific factor, residual stage-related effects likely remain and may still influence the observed factor patterns and loadings.

## Conclusion

By capturing the extent to which individuals express multiple overlapping tau deposition patterns, we offer a representation of disease biology that moves beyond traditional categorical frameworks. This approach provides a way to quantify the considerable heterogeneity observed in tau patterns, without losing nuance by applying rigid subtyping into mutually exclusive subgroups. Furthermore, as tau PET imaging becomes increasingly adopted in both research and clinical trials, individual-level characterization of tau spatial patterns may help improve precision in predicting cognitive measures, tailoring interventions, and enriching clinical trial cohorts. Ultimately, shifting from discrete diagnostic labels to continuous biological profiling will be essential for personalizing diagnostics and prognostics, and to tailor the next generation of targeted therapies to the individual.

## Supplementary Information

Supplementary Material 1.
