# Supplementary material for: Bayesian modelling demonstrates clinically relevant heterogeneity in Tau PET patterns in Alzheimer’s disease
Source: Eur J Nucl Med Mol Imaging. 2026 Mar 31;53(7):4664–76. doi: 10.1007/s00259-026-07868-5 (PMC13197381; doi:10.1007/s00259-026-07868-5)
Supplement: Supplementary file 1 — Supplementary Material 1. [file 259_2026_7868_MOESM1_ESM.docx]

**Supplement**

| **Transition** | **Δ log likelihood** | **Relative gain** |
| --- | --- | --- |
| K2 → K3 | +5.80 × 10⁶ | **0.45%** |
| K3 → K4 | +4.60 × 10⁶ | **0.36%** |
| K4 → K5 | +3.10 × 10⁶ | 0.24% |
| K5 → K6 | +2.35 × 10⁶ | 0.18% |

**Supplemental Table 1. Log likelihood gains with increased model complexity (number of factors [K])**

|  | **Amsterdam dementia cohort (ADC)** | | | |  |
| --- | --- | --- | --- | --- | --- |
| Factor | Baseline | | | |  |
|  |  | **Cognitive domain** | **Beta** | **SE** | **p** |
| 1 | Limbic | Memory | 0.03 | 0.14 | 0.81 |
|  |  | Executive functioning | 0.31 | 0.13 | 0.02 |
|  |  | Language | 0.27 | 0.13 | 0.04 |
|  |  | Visuospatial ability | -0.11 | 0.13 | 0.39 |
|  |  | MMSE | 0.23 | 0.12 | 0.05 |
|  |  |  |  |  |  |
| 2 | Left TPC | Memory | 0.04 | 0.11 | 0.73 |
|  |  | Executive functioning | 0.03 | 0.11 | 0.81 |
|  |  | Language | 0.08 | 0.12 | 0.51 |
|  |  | Visuospatial ability | 0.25 | 0.10 | 0.02 |
|  |  | MMSE | 0.12 | 0.10 | 0.25 |
|  |  |  |  |  |  |
| 3 | Posterior | Memory | -0.16 | 0.12 | 0.17 |
|  |  | Executive functioning | -0.11 | 0.12 | 0.35 |
|  |  | Language | -0.19 | 0.13 | 0.13 |
|  |  | Visuospatial ability | -0.21 | 0.11 | 0.07 |
|  |  | MMSE | -0.27 | 0.11 | 0.01 |
|  |  |  |  |  |  |
| 4 | MTL-sparing | Memory | 0.09 | 0.11 | 0.41 |
|  |  | Executive functioning | -0.14 | 0.11 | 0.19 |
|  |  | Language | -0.10 | 0.12 | 0.39 |
|  |  | Visuospatial ability | 0.06 | 0.11 | 0.62 |
|  |  | MMSE | -0.04 | 0.10 | 0.67 |

|  | **ADNI** | | | |  |
| --- | --- | --- | --- | --- | --- |
| Factor | Baseline | | | |  |
|  |  | **Cognitive domain** | **Beta** | **SE** | **p** |
| 1 | Limbic | Memory | 0.26 | 0.05 | 0.00 |
|  |  | Executive functioning | 0.34 | 0.06 | 0.00 |
|  |  | Language | 0.21 | 0.06 | 0.00 |
|  |  | Visuospatial ability | 0.24 | 0.07 | 0.00 |
|  |  | MMSE | 0.19 | 0.07 | 0.01 |
|  |  |  |  |  |  |
| 2 | Left TPC | Memory | -0.21 | 0.05 | **0.00** |
|  |  | Executive functioning | -0.11 | 0.07 | 0.09 |
|  |  | Language | -0.13 | 0.06 | **0.03** |
|  |  | Visuospatial ability | -0.03 | 0.07 | 0.70 |
|  |  | MMSE | -0.14 | 0.06 | **0.03** |
|  |  |  |  |  |  |
| 3 | Posterior | Memory | -0.05 | 0.05 | 0.32 |
|  |  | Executive functioning | -0.24 | 0.06 | 0.00 |
|  |  | Language | -0.07 | 0.06 | 0.21 |
|  |  | Visuospatial ability | -0.17 | 0.07 | 0.01 |
|  |  | MMSE | -0.01 | 0.06 | 0.82 |
|  |  |  |  |  |  |
| 4 | MTL-sparing | Memory | 0.05 | 0.06 | 0.33 |
|  |  | Executive functioning | 0.07 | 0.07 | 0.30 |
|  |  | Language | 0.03 | 0.06 | 0.57 |
|  |  | Visuospatial ability | 0.03 | 0.07 | 0.65 |
|  |  | MMSE | 0.04 | 0.07 | 0.50 |

|  | **ADNI** | | | |  |
| --- | --- | --- | --- | --- | --- |
| Factor | Longitudinal | | | |  |
|  |  | **Cognitive domain** | **Beta** | **SE** | **p** |
| 1 | Limbic | Memory | 0.04 | 0.02 | 0.01 |
|  |  | Executive functioning | -0.01 | 0.02 | 0.63 |
|  |  | Language | 0.01 | 0.02 | 0.62 |
|  |  | Visuospatial ability | 0.00 | 0.03 | 0.92 |
|  |  | MMSE | 0.03 | 0.03 | 0.33 |
| 2 | Left TPC | Memory | -0.12 | 0.02 | 0.00 |
|  |  | Executive functioning | -0.09 | 0.02 | 0.00 |
|  |  | Language | -0.13 | 0.03 | 0.00 |
|  |  | Visuospatial ability | -0.04 | 0.04 | 0.26 |
|  |  | MMSE | -0.20 | 0.03 | 0.00 |
| 3 | Posterior | Memory | -0.03 | 0.03 | 0.29 |
|  |  | Executive functioning | -0.02 | 0.03 | 0.50 |
|  |  | Language | -0.03 | 0.03 | 0.41 |
|  |  | Visuospatial ability | -0.11 | 0.05 | 0.02 |
|  |  | MMSE | -0.07 | 0.04 | 0.07 |
| 4 | MTL-sparing | Memory | 0.11 | 0.02 | 0.00 |
|  |  | Executive functioning | 0.11 | 0.02 | 0.00 |
|  |  | Language | 0.15 | 0.03 | 0.00 |
|  |  | Visuospatial ability | 0.09 | 0.04 | 0.01 |
|  |  | MMSE | 0.24 | 0.03 | 0.00 |

**Supplemental Table 2. Associations between factor loadings and cognition**

Associations between tau factor loadings and cognitive performance at baseline and longitudinally. Each table includes standardized beta estimates and standard errors from linear regression analyses (for baseline effects) or linear mixed-effects models (for longitudinal effects). Baseline effects were inferred from the fixed effect of factor on cognition, while longitudinal effects were inferred from factor*time=cognition interaction effects. Models were corrected for age, sex, education and syndromic diagnosis. TPC=temporoparietal cortex, MTL=medial temporal lob

|  | Age (Years) | | Sex (Male vs. Female) | | APOE ϵ4 (Carrier vs. NC) | |
| --- | --- | --- | --- | --- | --- | --- |
| Factor | β [95% CI] | p | β [95% CI] | p | β [95% CI] | p |
| ADC Cohort | | | | | | |
| Posterior | -0.01 [-0.02, -0.01]*** | <.001 | 0.11 [-0.00, 0.22] | 0.06 | -0.02 [-0.15, 0.10] | 0.74 |
| Left TPC | 0.00 [-0.01, 0.01] | 0.81 | -0.06 [-0.16, 0.05] | 0.28 | -0.03 [-0.14, 0.08] | 0.55 |
| MTL-sparing | -0.00 [-0.01, 0.00] | 0.28 | 0.07 [-0.05, 0.18] | 0.26 | -0.04 [-0.16, 0.09] | 0.59 |
| Limbic | 0.02 [0.01, 0.03]*** | <.001 | -0.12 [-0.23, -0.02]* | 0.03 | 0.09 [-0.02, 0.21] | 0.13 |
|  |  |  |  |  |  |  |
| ADNI Cohort | | | | | | |
| Posterior | -0.00 [-0.01, 0.00] | 0.45 | -0.04 [-0.12, 0.04] | 0.31 | 0.05 [-0.03, 0.13] | 0.24 |
| Left TPC | 0.00 [-0.01, 0.01] | 0.76 | -0.01 [-0.11, 0.10] | 0.93 | 0.04 [-0.07, 0.15] | 0.5 |
| MTL-sparing | -0.00 [-0.00, 0.00] | 0.84 | -0.01 [-0.04, 0.02] | 0.56 | -0.03 [-0.07, 0.00] | 0.07 |
| Limbic | 0.00 [-0.01, 0.01] | 0.76 | 0.06 [-0.05, 0.17] | 0.3 | -0.05 [-0.16, 0.06] | 0.34 |

Supplementary Table 3 Association between Demographics and Tau Factor Loadings

Table 2 are presented as unstandardized beta coefficients (β) with 95% confidence intervals (95% CI) and p-values derived from multivariate General Linear Models (GLM). For age, β represents the change in factor loading per 1-year increase in age. For sex, β represents the adjusted mean difference for males compared with females (reference group). For APOE, β represents the adjusted mean difference for ε4 carriers compared with non-carriers (reference group). Asterisks indicate statistical significance (*p < 0.05, **p < 0.01, ***p < 0.001).


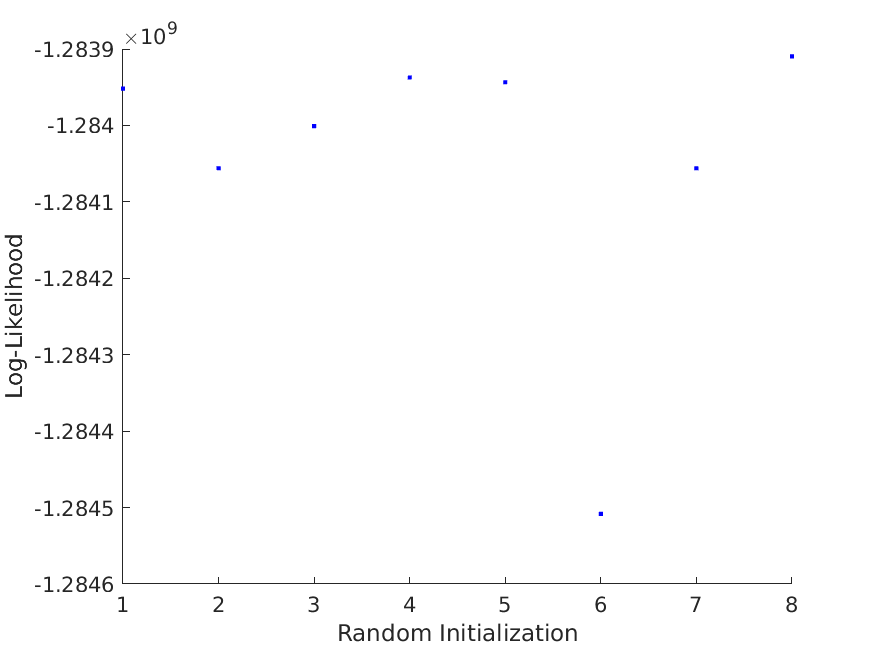


Supplemental Figure 1. Model fit across initializations of the LDA model (K=4).


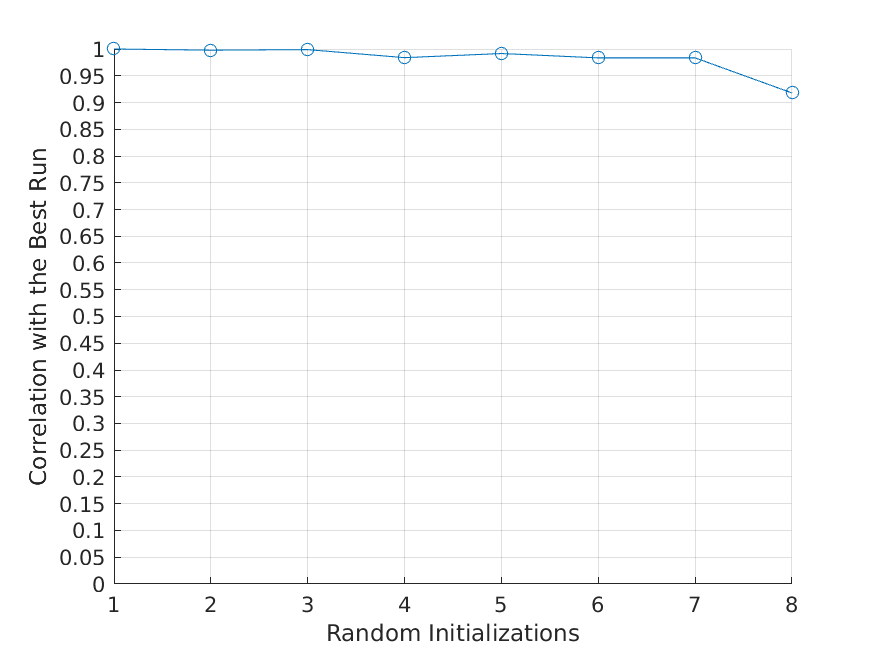


Supplemental Figure 2. Correlations between initializations of the LDA model (K=4).

References used in supplement

1. Groot C, Van Loenhoud ACAC, Barkhof F, et al. Differential effects of cognitive reserve and brain reserve on cognition in Alzheimer disease. Neurology 2018;90:e149-e156.

2. Zhang X, Mormino EC, Sun N, Sperling RA, Sabuncu MR, Yeo BT. Bayesian model reveals latent atrophy factors with dissociable cognitive trajectories in Alzheimer's disease. Proc Natl Acad Sci U S A 2016;113:E6535-e6544.
